# Distinct Effects of Memory Retrieval and Articulatory Preparation when Learning and Accessing New Word Forms

**DOI:** 10.1371/journal.pone.0126652

**Published:** 2015-05-11

**Authors:** Anni Nora, Hanna Renvall, Jeong-Young Kim, Elisabet Service, Riitta Salmelin

**Affiliations:** 1 Department of Neuroscience and Biomedical Engineering, Aalto University, Espoo, Finland; 2 Aalto NeuroImaging, Aalto University, Espoo, Finland; 3 Department of Neurology, Helsinki University Central Hospital, Helsinki, Finland; 4 Department of World Cultures, University of Helsinki, Helsinki, Finland; 5 Department of Linguistics and Languages, McMaster University, Hamilton, Canada; 6 Institute of Behavioural Sciences, Division of Cognitive and Neuropsychology, University of Helsinki, Helsinki, Finland; University of Barcelona, SPAIN

## Abstract

Temporal and frontal activations have been implicated in learning of novel word forms, but their specific roles remain poorly understood. The present magnetoencephalography (MEG) study examines the roles of these areas in processing newly-established word form representations. The cortical effects related to acquiring new phonological word forms during incidental learning were localized. Participants listened to and repeated back new word form stimuli that adhered to native phonology (Finnish pseudowords) or were foreign (Korean words), with a subset of the stimuli recurring four times. Subsequently, a modified 1-back task and a recognition task addressed whether the activations modulated by learning were related to planning for overt articulation, while parametrically added noise probed reliance on developing memory representations during effortful perception. Learning resulted in decreased left superior temporal and increased bilateral frontal premotor activation for familiar compared to new items. The left temporal learning effect persisted in all tasks and was strongest when stimuli were embedded in intermediate noise. In the noisy conditions, native phonotactics evoked overall enhanced left temporal activation. In contrast, the frontal learning effects were present only in conditions requiring overt repetition and were more pronounced for the foreign language. The results indicate a functional dissociation between temporal and frontal activations in learning new phonological word forms: the left superior temporal responses reflect activation of newly-established word-form representations, also during degraded sensory input, whereas the frontal premotor effects are related to planning for articulation and are not preserved in noise.

## Introduction

Learning a novel language in adulthood starts with the hearing and encoding of novel word forms. Novel phonological forms, even without a known meaning, can be learned incidentally when encountered multiple times, especially when overt reproduction is attempted [1–3]. The ability to accurately repeat meaningless verbal sequences, a measure of working memory capacity, has been established as an important probe of language learning abilities in both first and second language [4,5], and it is especially predictive of learning new vocabulary [6,7]. Therefore, in order to understand individual and age differences in language acquisition, the neural mechanisms involved in processing and learning novel phonological forms without meanings should be addressed.

At the brain level, both hemodynamic (functional magnetic resonance imaging, fMRI; positron emission tomography, PET) and electrophysiological (magnetoencephalography, MEG) measures have consistently identified decreased activation in the left superior temporal cortex as a marker of within-session learning for novel, repeatedly presented word forms of the native phonology [8–11]. Changes in frontal activation have been reported as well, but they have been more variable [8,11–14]. For example, decreased hemodynamic responses have been observed in the premotor and left inferior frontal cortices for repeated word forms [11,12]. However, in a recent time-sensitive MEG study, repeated pronunciation of foreign phonological forms (Korean words) and new native-language word forms (Finnish pseudowords) resulted in an increased frontal activation at 600–1200 ms after the model stimulus onset. Coactivity of temporal and frontal regions along the dorsal route of speech processing has been linked to binding of auditory and articulatory information for sensory-motor representations [15,16], but the specific roles of these areas in learning novel word forms are not known. The involvement of the frontal cortex in phonological learning is especially interesting, since activation in premotor regions has been linked to speech processing, but much controversy exists as to what its role precisely is (for reviews see [17–21]).

Frontal activation prior to overt repetition has been suggested to reflect phonological planning for articulation [22–24]. However, recent results suggest that left frontal activation for newly learned word forms may be related to formation and retrieval of articulatory representations, rather than to articulatory planning. Such activation was observed in a post-learning test phase even when no articulation was required, provided that the preceding learning phase had involved overt repetition [16]. According to some studies, frontal premotor areas are involved in speech perception mainly if detailed motor representations are subsequently needed to perform an action [25], or when the task requires detailed parsing or segmentation of the phonetic structure of the incoming signal [26–30]. The motor system has been suggested to be recruited especially during effortful speech processing, for instance, when the auditory signal is degraded or when processing a foreign language. Activation of the premotor cortex has been reported to be especially pronounced during speech processing in demanding listening conditions [31,32]. Transcranial magnetic stimulation of the premotor cortex has also been reported to impair phonetic discrimination in noise [33], suggesting that motor representations are utilized to facilitate speech perception in conditions with insufficient bottom-up information. These studies have focused mainly on perception at the phonetic level. Motor processes might be involved mainly in tasks requiring explicit phonological judgments [34], although according to some views premotor regions also might have a role in word-level comprehension [35]. It is possible that the premotor effects observed for newly-learned word-form level units would reflect retrieval of word-form level articulatory representations to support auditory perception. In the present study we set out to investigate whether the frontal learning effect merely results from planning for overt articulation of recently learned word forms, or whether it also has a role in perception, which would, presumably, be more pronounced in demanding listening conditions.

In the current MEG study, we employed various post-learning manipulations to illuminate the roles of the temporal and frontal cortices in phonological processing, with the frontal activation as our particular interest. In speech processing experiments, premotor cortical activation is typically not observed without a specific task that would engage the motor system [15,19,36] (but see [37]). Nevertheless, motor involvement is often reported in the context of word learning [14,38–40], even with a passive listening task [13], suggesting that during learning of novel auditory word forms the auditory input is automatically linked to motor representations, possibly through covert rehearsal (for a review see [41]). In the current experiment, to ensure the initial engagement of the motor system while minimizing the influence of semantics, we utilized incidental learning of meaningless word forms in an overt repetition task. Constructing sensory-motor representations for new phonological sequences appears to critically depend on coactivity of auditory and motor systems [38,39,42] and has been shown to engage both temporal and frontal regions during input processing [10]. As acquisition of new phonological forms is easier if the stimuli adhere to the phonotactic structure of the native language, we included stimuli with a familiar (native) and a novel (foreign) phonotactic structure. This allowed us to manipulate the availability of familiar phonotactic (phoneme sequencing) regularities in processing.

The current experiment began with an initial learning phase comparable to that described by Nora et al. [10] which had allowed to identify functionally distinct responses to novel phonological forms in the superior temporal cortex (familiar < new word forms) and frontal premotor cortex (familiar > new word forms). Participants listened to and repeated back foreign phonological forms (Korean words) and new native-language word forms (Finnish pseudowords) that were encountered four times during the learning phase. This phase served as a functional spatio-temporal localizer for cortical areas and time windows that play a salient functional role in word-form level speech processing. Subsequently, we focused on the cortical responses that showed learning effects.

After the learning phase, participants were exposed to four experimental conditions, each with the familiar stimuli from the learning phase mixed with completely new word forms. The experiment consisted of two overt repetition conditions with different levels of added stimulus noise, and two conditions with different tasks. To delineate the role of frontal response reactivity in overt articulation, the need for articulatory translation was varied in two tasks. A 1-back control task combined with overt production of a repeated control word was administered: such a task required auditory processing and memory maintenance of the items but prevented formation of item-specific gestural scores for articulation as the produced control word was always the same. At the end of the session, the participants performed a surprise recognition task requiring receptive and recognition processes but no overt articulation. If the frontal learning effects reflect memory retrieval, they should be observed in all three tasks. In contrast, effects that are directly related to articulation should show up only in the conditions involving overt item repetition.

Parametric addition of noise was used to highlight effects reflecting retrieval of established word-form representations. Models of speech processing (e.g. [43]) propose that the influence of prior knowledge, in this case the established word-form level phonological representations, is greatest when the speech signal is degraded. We hypothesized that the effects of word-form familiarity in the temporal and/or frontal cortices would be more pronounced during speech perception in noise, reflecting increased influence of existing (auditory or motor) word-form level representations on online speech processing. This would follow from reduced availability of sensory information necessitating reliance on existing representations for accurate perception and reproduction of items (see e.g. [44]). The frontal effects might be particularly emphasized here, since articulatory processes have been suggested to facilitate perception in noise [31,32,45,46]. Furthermore, we expected noise-induced enhancement of the word-form familiarity effects to be particularly strong for the native language stimuli, with familiar phonotactic frames available to support learning and retrieval.

## Materials and Methods

### Participants

We recorded data from 12 right-handed, Finnish-speaking adults (5 females and 7 males; age 20–42 years, mean 25 years) with no previous experience of Korean language. Participants’ handedness was established with an adapted version of the Edinburgh Handedness Inventory [47]. All participants had normal hearing and no diagnosed neurological or language disorders. All participants gave their written informed consent to participate in this study, in agreement with the prior approval of the Aalto University Research Ethics Committee.

### Experimental stimuli

The stimuli were taken from a previous study [10]. They consisted of 400 four-syllable real Korean words or word combinations, and 400 four-syllable Finnish pseudowords composed of two-syllable Finnish words no longer in use [48]. The duration of the words varied from 861 to 1446 ms (mean 1130 ms) for Korean and from 918 to 1407 ms (mean 1240 ms) for Finnish, without statistically significant difference in mean duration between stimulus conditions.

The Korean words were all selected and digitally recorded by a female native Korean linguist, speaking standard Korean. The Finnish pseudowords were selected by the authors and recorded by a female native Finnish speech pathology student. All stimuli began with consonants, and stimuli in both languages contained equal numbers of nasal, fricative/affricate, and stop consonants as initial consonants in each stimulus category. The first syllable was frequently shared between two or more words, with isolation points at the second syllable, at 250–300 ms.

The words were recorded in 24-bit wav format using a sampling rate of 48 kHz and, to eliminate background noise, low-pass filtered with a cutoff frequency of 6 kHz. Stimuli were equalized, as much as possible, with respect to acoustic properties that are known to have an influence on early auditory cortical responses, such as stimulus intensity (average RMS over stimulus duration) and rise time [49,50]. A 10-ms ramp was added to the beginning and end of each word to avoid clipping.

Ten participants performed a behavioral pilot study with the Finnish and Korean stimuli to quantify the effect of added background noise on detection of immediate stimulus repetitions in a 1-back task. The signal-to-noise ratios (SNRs) of +20 dB, +10 dB, 0 dB, and—10 dB were achieved by embedding the stimuli in different levels of white noise (low-pass filtered at 6 kHz) and keeping the overall root-mean-square amplitude value unchanged. At SNR +20 dB, +10 dB and 0 dB the sounds were easy to distinguish, supported by perfect performance in the 1-back task. Because task accuracy began to deteriorate at SNR—10 dB, SNRs of +10 dB and 0 dB were selected to be included in the MEG study, in addition to the noiseless stimuli (SNR +70 dB).

To further characterize the acoustic properties of the Finnish and Korean speech sounds, sound intensity envelopes, harmonics-to-noise ratios (HNRs) and sound spectra were examined in more detail with the freely available Praat software (www.fon.hum.uva.nl/praat/). Sound intensities were analyzed in 50-ms steps from the beginning of the stimuli. The intensity of the stimuli reached a maximum at 100–150 ms for Finnish word forms and at 200–250 ms for Korean word forms, reflecting differences in the stress patterns between the two languages (stimulus intensity was significantly stronger for Finnish in the 0–250 ms time window, P < 0.01). HNR reflects the degree of periodicity of the sound [51], and it is quantified as the energy of the periodic (harmonic) component of the signal over time relative to the remaining ‘‘noise” signal. The obtained HNRs were generally low for all stimuli because of the silent periods in the stimuli. HNRs for Korean and Finnish stimuli were within normal limits (> 10 dB) for our female speakers when silent bits were removed, but—as the stimuli were produced by one speaker for each language—the difference between languages remained statistically significant. The same was true for frequency spectra of the sounds, estimated in 50-Hz frequency steps. Although the Finnish and Korean stimuli, as such, could not be fully matched acoustically, the parametric addition of noise was identical for both languages.

### Experimental procedure

**Fig 1:**
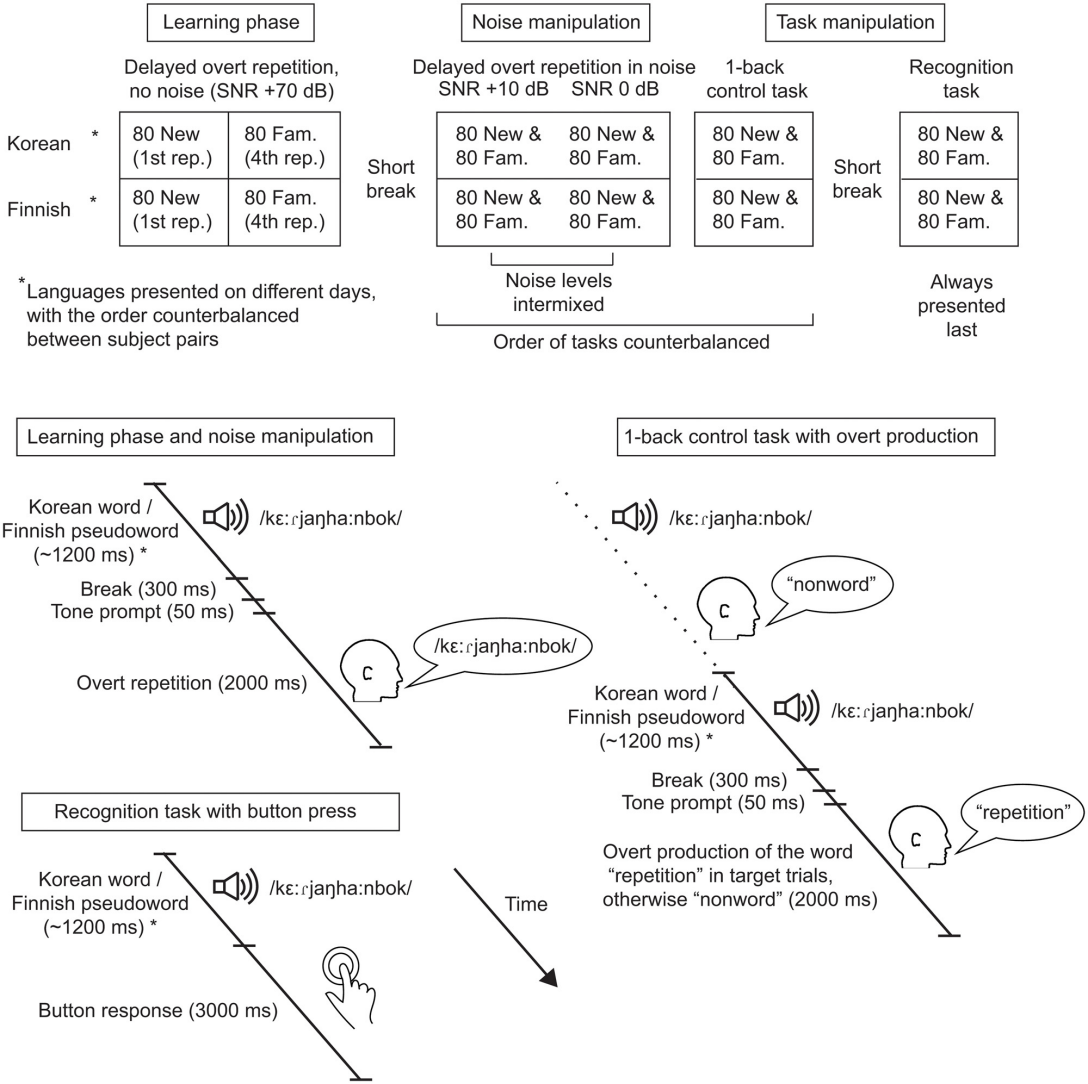
Experimental design. The initial learning phase with overt repetition was followed by two experimental manipulations, through noise and task. *The noise manipulation* included overt repetition of familiar and new word forms in two levels of noise. *The task manipulation* consisted of a 1-back control task and a recognition task that were performed on familiar and new word form stimuli. Stimulus presentation and response timing in the different experimental conditions is illustrated below.

The experimental design is illustrated in Fig 1. Participants performed the same tasks in native and foreign language in separate sessions, with the order of the languages counterbalanced between participants. In the initial learning phase, participants heard and repeated 80 words that were each presented four times. Presentation of the stimuli consisted of four blocks, each containing one presentation of each item. The experimental setup of the learning phase was comparable to our previous study that showed significant learning effects [10]. However, in order to accommodate the multiple post-learning test conditions while keeping the entire experiment duration tolerable, the paradigm was modified slightly: all stimuli recurred four times (instead of five times as in the original study) and there were fewer stimuli per stimulus group (80 instead of 100). The current study did not include the category of nonrecurring items presented only once during the learning session. Instead of comparing nonrecurring and recurring stimuli as in our previous study [10], we used comparison of the first and last repetition of the recurring items as a measure of learning. In the Nora et al. [10] study, the differences between first and last repetitions were similar to those between recurring and nonrecurring items.

The participant’s task was to listen to the words and, after 300 ms of silence and a 50-ms tone prompt, to repeat them as accurately as possible. Delayed repetition was used to avoid contamination of the MEG signals by muscle activity. Participants had 2 s to repeat the heard word form. Participants were not instructed to memorize or learn the words in any way, only to repeat them as accurately as possible. The participants’ repetition responses were recorded and later evaluated for accuracy.

After the learning phase and a break of ~15 minutes, participants encountered four experimental conditions: two with overt repetition of the items embedded in noise (SNR +10 dB and 0 dB), a 1-back control task (no noise) and a surprise recognition task (no noise). Each of the four post-learning conditions included one presentation of the familiar stimuli used in the learning phase (80) mixed with a completely new set of 80 items per condition (Fig 1). The stimuli at the two noise levels were presented intermixed together. The order of the repetition task in noise and the 1-back control task was balanced between participants. The recognition task was always presented last.

In the noise conditions, the participants performed a delayed overt repetition task, similarly as in the learning phase: The participant’s task was to listen to the words and, after 300 ms of silence and a 50-ms tone prompt, to repeat them as accurately as possible. In the 1-back control task, the presentation of the noiseless familiar and new stimuli was similar to that in the learning phase. The participant’s task was to listen to the words and, after 300 ms of silence and a 50-ms tone prompt following each stimulus, to produce one of two pre-specified response words. In target trials (20 of the 80 familiar items and 20 of the 80 new items), the same item was presented twice in a row, and participants were instructed to respond by producing the word “toisto” (“repetition”). After non-target words, they always produced the four-syllable standard word “epäsana” (“nonword”). Participants had 2 s to respond. Target trials (with “repetition” responses) were excluded from analysis.

At the end of the session, participants completed a recognition test, which they were not warned about beforehand. Participants now heard the newly learned words (80) from the learning phase (without noise) mixed with a similar number of new words. The participants' task was to indicate by a button press for each word, as fast as possible, if it had been presented before. The participants used one hand to respond yes and the other to respond no; the response hands were alternated across participants. The participant had, on average, 3000 ms to respond (SOA from onset to onset was always 4200 ms). The participants’ responses were scored for accuracy, and their reaction times were measured.

In the following, “new words” refers both to the first presentation of the recurring words in the learning task and to the words that were introduced for the first time in each of the post-learning tasks. The expression “familiar words” refers to the fourth presentation of the recurring words in the learning phase as well as to this same group of words presented again in the post-learning tasks. To control for the variation in difficulty between individual stimuli, the recurring and new words were counterbalanced between participant pairs, and the sets of new stimuli were rotated across tasks.

### MEG recordings

The MEG recordings in the learning phase lasted ~20 minutes, in the noise conditions ~10 minutes, in the 1-back control task ~5 minutes, and in the recognition task ~10 minutes. Participants were instructed to keep their eyes open and fixate on a mark on the wall before them. Hearing threshold was determined individually for each participant at the start of the session, and the stimuli were delivered through plastic tubes and earpieces at 70 dB (sensation level). Participants’ oral responses (in the repetition tasks) were recorded with a digital tape recorder. In the recognition task, manual responses were collected with an optical response device.

The magnetic fields associated with neural current flow were recorded in a magnetically shielded room with a 306-channel whole-head neuromagnetometer (Elekta Oy, Helsinki) at the Aalto NeuroImaging MEG Core. During data acquisition, the MEG signals were band-pass filtered between 0.03 and 200 Hz and sampled at 600 Hz. Horizontal and vertical electro-oculograms (EOGs) and an electromyogram (EMG) were recorded to discard data contaminated by blinks and eye and mouth movements.

The position of the participant’s head within the MEG helmet was determined using four head position indicator coils. The locations of these coils, attached to the participant’s scalp, were determined with respect to three anatomical landmarks (nasion and two preauricular reference points) with a 3D digitizer and to the sensor array by briefly feeding current to the coils before the actual measurement.

### Anatomical magnetic resonance imaging

Anatomical magnetic resonance images (MRIs) were obtained for all participants with a 3T MRI scanner (Magnetom Skyra, Siemens) in a separate session after the MEG measurements. The scan included a 3-plane localizer and two T1-weighted anatomical images. The MEG data were co-registered in the same coordinate system with the individual anatomical MR images to allow construction of the head conductor model for the MEG source-level analysis and to enable visualization of the MEG-derived activation patterns on the brain structure.

### Behavioral analysis

Accuracy scoring followed the typical procedure in pseudoword repetition tasks. The overt repetitions produced by the participants during the first and the last blocks of the learning phase and during post-learning noisy repetition conditions, were rated in a scrambled order by native Korean and Finnish speakers, for the two languages respectively; these evaluators were unaware of the experimental manipulations. One point was given for each whole word that had been accurately repeated (none of the phonemes omitted, replaced with other phonemes or transposed). However, phonetically native-like articulation or prosody was not required, and detailed evaluation of acoustic-phonetic deviations was outside the scope of this study. Somewhat deviant or “accented” output was accepted as correct reproduction, following the practice in foreign word repetition research [52,53]. The delayed response procedure prevented us from recording response latencies during the repetition tasks. In the recognition task, response accuracy was recorded.

### MEG data analysis

The MEG signals were averaged from 200 ms before to 1200 ms after the stimulus onset, rejecting trials contaminated by eye movement, blink or mouth movement artifacts (percent of accepted trials 80 ± 13.4%, mean ± standard deviation). The averaged MEG responses were baseline-corrected to the 200-ms interval immediately preceding the stimulus onset, and digitally low-pass filtered at 40 Hz. In addition, because of the variance in stimulus durations, MEG signals were also averaged with respect to the EMG signal, but this did not change the results significantly. The analysis began with a sensor-level visual inspection of the results. A source-level overview of the spatiotemporal distribution of neural activity was obtained by minimum norm estimates (MNEs) [54] using the MNE Suite software package (Martinos Center for Biomedical Imaging, Massachusetts General Hospital) [55]. MNE implements the cortically-constrained L_2_ minimum-norm estimate of the source distribution, which aims to identify the current distribution that explains the measurements and has the lowest overall power. MNE analysis results in distributed models of the cortical activation; however, the resulting maps do not indicate the actual shape or extent of the activated areas.

For MNE analysis, the cortical surface of each participant was reconstructed from their individual MR images with the Freesurfer software [56,57]. Each hemisphere was covered with ~5000 potential source locations. Currents oriented normal to the cortical surface were favored by weighting the transverse currents by a factor of 0.2, and depth-weighting was used to reduce the bias towards superficial sources [58]. Noise-normalized MNEs (dynamical Statistical Parametric Maps, dSPMs) were calculated over the whole cortical area to estimate the signal-to-noise ratios in each potential source location [59]. A noise covariance matrix was estimated from the 200-ms prestimulus baseline periods in the raw data. For group-level visualization, the individual MNEs were first normalized to the maximum value of each participant across conditions and subsequently morphed, with spatial smoothing, to a standard brain.

The MNE method does not distinguish between multiple independent sources that are spatially proximate but have different dominant orientations of current flow, typical of auditory word processing [60,61]. Therefore, separable cortical-level spatiotemporal components were estimated by means of Equivalent Current Dipole modeling (ECD) [62]. Sensor selection for the ECD modelling was limited to the planar gradiometers. Only ECDs explaining more than 80% of the local field variance during their peak activation were accepted in the model. This criterion led to the inclusion of 5–7 ECD components per participant; for most participants, two active source areas could be identified in each temporal cortex and one in each frontal cortex. A single component in the temporal cortex accounted well for the (similar) field patterns around 150 and from 400 ms onwards, and a separate component explained the current flow at ~250 ms. A separate model was first identified for each experimental condition. A single set of ECDs explained well the responses in all conditions and was thus used to allow comparison between conditions. The time courses of the identified spatiotemporal components (source waveforms) were estimated by fixing their location and orientation parameters while allowing their strengths to vary to best account for the signals detected by all MEG sensors over the entire analysis interval. To locate the ECD components anatomically, the center of activation of each component was displayed on the individual MRIs of each participant. For group-level visualization, the locations were transformed to a standard brain [63]. The ECD location parameters were first transformed into the Montreal Neurological Institute (MNI) reference space [64], and approximation of Talairach coordinates [65] was achieved by linear conversion.

### Statistical analysis

Behavioral repetition accuracy was first analyzed for the new word forms (in the 1^st^ block) and familiar words (in the 4^th^ block) of the learning phase using a repeated-measures 2 x 2 (Language x Stimulus familiarity) ANOVA. To analyze how repetition accuracy was affected by noise, the new and familiar words of the learning phase, as well as the new and familiar words embedded in the two levels of noise, were entered into a repeated-measures 2 x 2 x 3 (Language x Stimulus familiarity x Noise level) ANOVA. Further planned pair-wise t-tests were Bonferroni corrected. Appearance of substitution errors in the repetition of native stimuli during the noise manipulation was evaluated by entering the percentage of substitutions with real words in each noise condition into a repeated-measures 2 x 3 (Stimulus familiarity x Noise level) ANOVA. For behavioral recognition results, a discriminability measure between the familiar and new words (d’) was calculated individually for each participant, taking into account the false alarm rates. This measure was compared between the two languages with a paired-samples t-test. When repetition and recognition accuracy results could not be obtained for all conditions for all participants because of technical problems, missing cases were removed listwise; this led to the inclusion of 8–11 participants in the analysis of repetition accuracy (11 in the analysis of the learning phase and 8 in the analysis on all noise levels) and 11 participants in the recognition accuracy analysis.

Statistical analysis of the cortical results was performed on the ECD source waveforms that were divided into a set of relevant time windows that covered the onset, peak and decline of the responses. The maximum of a transient increase of neural activity at ~150 ms in the type I temporal sources (see Results) was determined separately for each participant. A transient reduction of activity at ~200 ms (in the type I temporal sources) and an increase of activity at ~250 ms (in the type II temporal sources) were described by the mean source strengths within a 100-ms time window centered on the lowest and highest point, defined separately for each participant. For sustained effects, the same time windows were used for all participants. In the learning phase, the sustained temporal effect was divided into three 300-ms time windows, 300–600 ms, 600–900 ms and 900–1200 ms, describing the onset, plateau and decline of the responses, respectively, and the same time windows were used for the frontal responses to allow timing comparisons. When significant effects were found in several consecutive time windows of the sustained responses, those time windows were pooled together to simplify the description of the results. In the post-learning conditions, statistical testing was performed on the cortical areas and time windows that showed learning effects during the learning phase.

The group-level cortical analyses were conducted on the spatiotemporally congruent components of cortical activation (represented by the ECDs) that had a similar orientation and temporal evolution of current flow. Clusters that contained data from at least 6/12 participants were included in the statistical comparisons. For verifying possible learning effects, a repeated-measures 2 x 2 ANOVA was run with the factors of Language (native vs. foreign) and Stimulus familiarity (1^st^ vs. 4^th^ repetition). The influence of experimental task on the learning effect (in the noiseless conditions) was analyzed in a repeated-measures 2 x 2 x 3 ANOVA with the factors Language (native vs. foreign), Stimulus familiarity (familiar vs. new) and Task (repetition task without noise / 1-back control task / recognition task). In the recognition task, all trials were included in the averaged MEG signals; however, the results did not change when only correct trials (hits and correct rejections) were included in the analysis. For estimating the influence of noise level on the learning effect (in the repetition task), previously heard (familiar) and new stimuli from each noise level were included in a repeated-measures 2 x 2 x 3 ANOVA with the factors Language (native vs. foreign), Stimulus familiarity (familiar vs. new) and Noise level (+70 dB = no noise / SNR +10 dB / SNR 0 dB).

For evaluating the relationship between behavioral and cortical effects, Spearman’s pairwise correlations were computed between the neural learning effects and the improvement in repetition accuracy for the familiar words in intermediate noise as well as recognition accuracy. The improvement of repetition accuracy during learning was weak for Korean word forms and too near ceiling for Finnish word forms to allow calculation of correlations. A non-parametric test was used because of the low number of observations. The improvement in behavioral repetition accuracy was estimated as the percentage change in repetition accuracy of the familiar compared to new items, and success in recognition as the sensitivity index d’ that accounts for response bias. The neural measure was signal change in the learning phase from the 1^st^ to the 4^th^ repetition in the time windows and cortical sources showing learning effects, normalized to each participant’s initial activation level and expressed as percentage of the signal strength at the 4^th^ repetition with respect to the signal strength at the 1^st^ repetition.

## Results

### Behavioral results

**Fig 2:**
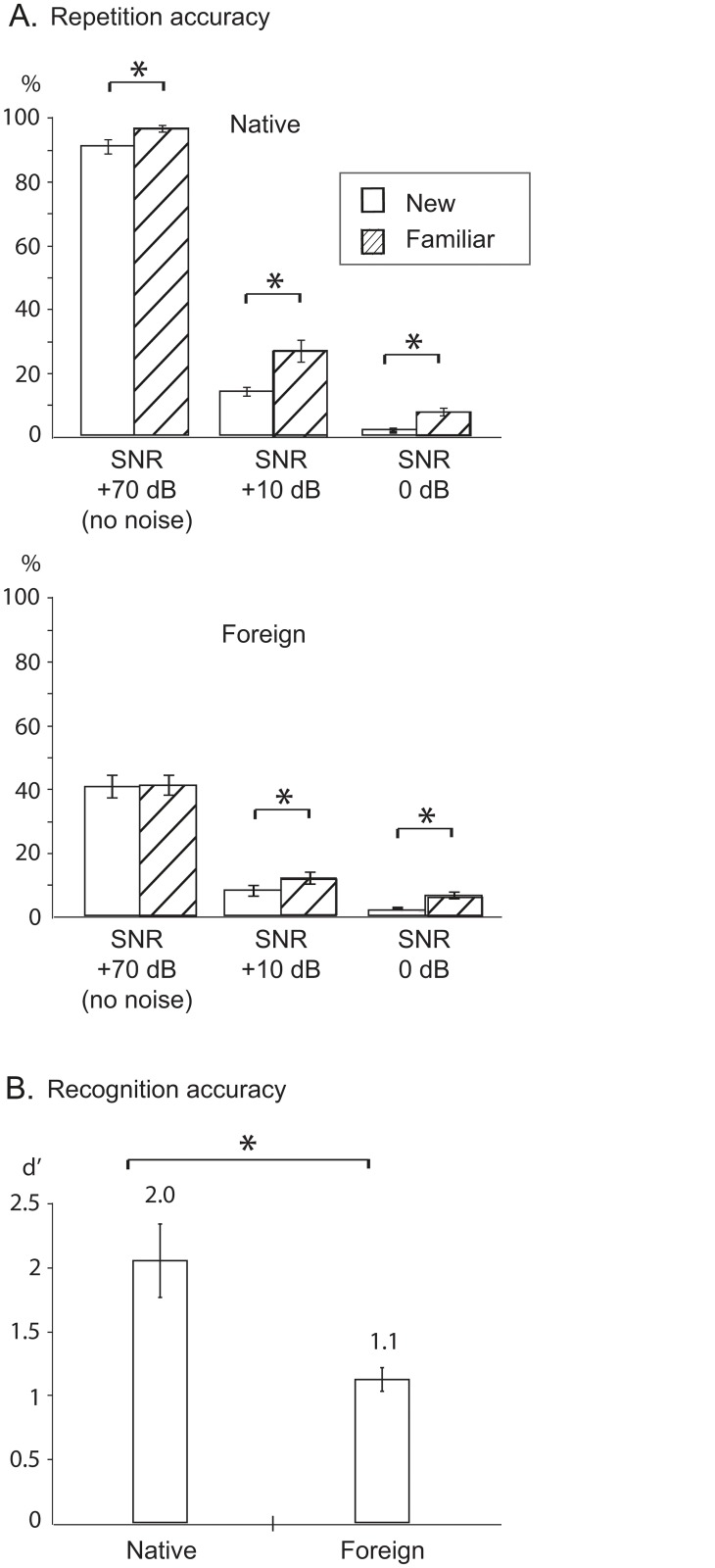
Behavioral effects. (A) Proportion of correctly repeated familiar and new foreign and native word forms (mean ± standard error of mean) in different levels of noise (signal-to-noise ratio = SNR +70 dB / +10 dB / 0 dB). * refers to p < 0.05. (B) Proportion of correctly recognized familiar foreign and native word forms (hits; mean ± standard error of mean).

In the learning phase participants correctly repeated on average 88% (new; 1st presentation) and 93% (familiar; 4th presentation) native word forms and 40% (new) and 41% (familiar) foreign word forms (Fig 2A). Repetition accuracy was significantly higher for the familiar word forms than new word forms [F(1,8) = 10.0, p < 0.05], and it was also better for the native than the foreign word forms [F(1,8) = 131.3, p < 0.01], but there was no significant interaction that might have suggested different learning effects for the two languages.

Added noise had a significantly detrimental effect on repetition performance [F(2, 6) = 413.4, p < 0.01]. At SNR +10 dB, participants correctly repeated on average 13% of the new and 26% of the familiar native word forms and 8% of the new and 12% of the familiar foreign words. At SNR 0 dB, performance was very low (1% and 7% for native language, 2% and 6% for foreign language new and familiar words, respectively) (Fig 2A). The main effect of Stimulus familiarity remained significant when all three noise levels were entered into the analysis [F(1,7) = 36.6, p < 0.01]. Paired comparisons revealed a significant benefit for familiar compared to new words at both non-zero noise levels [across languages; SNR +10 dB: t(8) = 4.6, p < 0.01; SNR 0 dB: t(8) = 5.3, p < 0.01; Bonferroni corrected alpha = 0.025]. The proportion of correctly repeated words (repetition accuracy) was significantly better for the native than for the foreign stimulus items also in noise [across noise levels; F(1,7) = 95.7, p < 0.01]. The results show a significant two-way interaction of Language and Noise level [F(2, 6) = 41.7, p < 0.01]: paired comparisons revealed better performance for native than foreign stimuli with no noise and at SNR +10 dB [across new and familiar words; no noise: t(8) = 11.4, p < 0.01; SNR +10 dB: t(8) = 7.0, p < 0.01; Bonferroni corrected alpha = 0.017], but this difference disappeared at SNR 0 dB [t(8) = 0.19, p = 0.86].

The number of substitutions with real words in the native language was relatively higher for the two noise conditions (SNR +10 dB: 6% and SNR 0 dB: 9%) than for the no-noise condition (1%) [main effect of Noise level: F(2,7) = 13.6, p < 0.01]. Overall, there were more substitution errors for familiar than unfamiliar stimuli [main effect of Stimulus familiarity: F(1,8) = 8.1, p < 0.05].

Of the words that had been encountered during the learning phase, the participants recognized 70% in the native language (53–98%; d’ = 2.0) and 57% in the foreign language (39–79%; d’ = 1.1) [native better than foreign; t(10) = 3.8, p < 0.01; Fig 2B].

### MEG results

**Fig 3:**
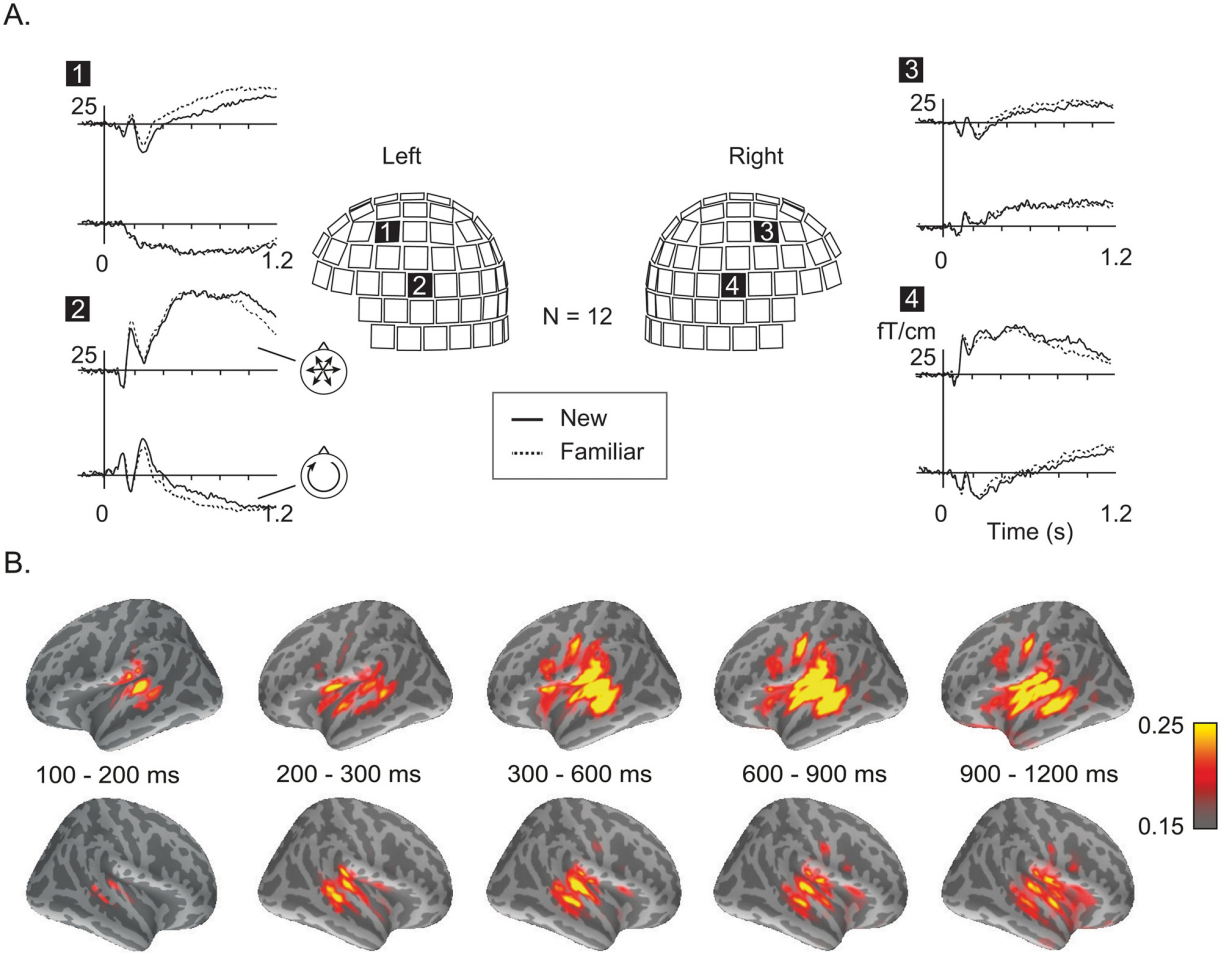
Sensor signals and source-level overview. (A) MEG sensor-level data. First (‘new’) and fourth (‘familiar’) presentation of the novel word forms in the learning phase, averaged over twelve participants and over languages. Stimulus effects are illustrated at two recording sites over the left hemisphere (sites 1, 2) and two (symmetrical) sites over the right hemisphere (sites 3, 4), with each site containing two planar gradiometers that are sensitive to orthogonal orientations of neural currents (cf. small schematic heads). Planar gradiometers detect the strongest signal directly above an active cortical area. (B) Source localization using minimum norm estimates (MNEs). The distribution of activation advancing from 100 to 1200 ms after stimulus onset (time slots of 100 or 300 ms), represented as an average over twelve participants. Salient activation maxima are evident in the bilateral temporal and left frontal cortices, but spatially close sources merge into a single blob, even when they have different underlying orientations of current flow.

At the sensor level, an overall similar response pattern was seen for all stimuli and task conditions. A prominent transient response was detected at about 150 ms, followed by a strong sustained response from about 300 ms onwards (Fig 3A, sensor-level responses in the learning phase) with source areas bilaterally in the superior temporal cortex as indicated by MNE analysis (Fig 3B). The frontal cortices also contributed to the measured sustained response, particularly in the left hemisphere.

**Fig 4:**
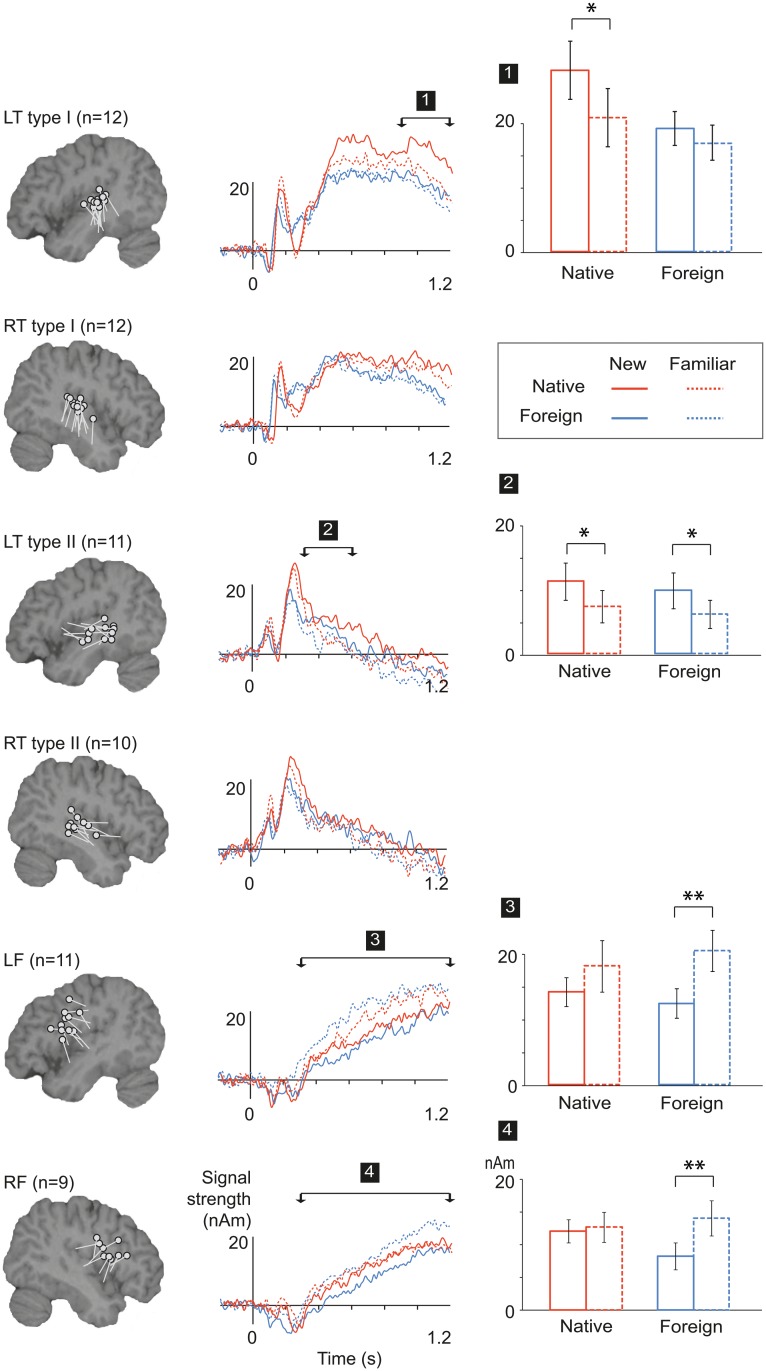
Group-level experimental effects in the learning phase. *Left*: Equivalent Current Dipole (ECD) clusters displayed on a sagittal plane of a standard brain. Each dot represents the center of an active cortical patch and the line attached to it the mean direction of current flow in that area, in one participant (LT = left temporal, RT = right temporal, LF = left frontal, RF = right frontal). *Center*: Grand average source waveforms for each ECD cluster, for new and familiar word forms in the native and foreign language. The arrows indicate the time windows that showed statistically significant effects (indexed by numbers 1 through 4). *Right*: Summary of the significant main effects and interactions of the mean activation strengths (cf. indices on the source time courses). Error bars indicate the standard error of mean. Asterisks denote statistically significant effects in paired comparisons of familiar and new word forms (* p < 0.05, ** p < 0.01).

ECD modeling allowed further decomposition of the activation within each temporal cortex into two separate sources of neural current flow. These sources could be distinguished both based on their temporal activation patterns and, at the level of individual participants, based on their approximately perpendicular orientations of neural current flow. “Type I” sources explained well both the early transient response (frequently referred to as N100m) and the sustained response peaking at ~400 ms, and their current flow was directed along the superior-inferior axis. “Type II” sources reached their maximum at ~250 ms, and they had a posterior-anterior direction of neural current flow (Figs 3A and 4). Based on the source locations in the individual MRIs, as well as on conversion to a common reference space, these two temporal source clusters resided in the Heschl’s gyrus or more posterior in the planum temporale (type I), and the posterior superior temporal sulcus (type II).

Type I temporal sources were identified bilaterally in all 12 participants. Type II temporal sources were found in 11/12 participants in the left hemisphere (LH) and in 10/12 participants in the right hemisphere (RH). Additionally, an active source area in the frontal cortex was detected in 11/12 participants in the left hemisphere and in 9/12 participants in the right hemisphere. In both hemispheres, these sources pointed to activation at or near the premotor cortex (inferior precentral sulcus / bordering on inferior frontal sulcus). In the following, we first identify cortical areas and time windows that manifested learning effects in the initial phase of learning by overt repetition. We then examine how those activations are influenced by tasks and added noise.

### Learning effects

Effects of learning (response changes from the 1^st^ to the 4^th^ repetition of the word forms during the overt repetition task) in different source clusters are depicted in Fig 4 for both languages. Source strengths in the time windows displaying learning effects are presented in Table 1. The earliest effects were observed as reduction of activity to familiar items in the LH temporal type II sources at 300–600 ms [F(1, 10) = 13.2, p < 0.01] (Fig 4, Table 1). Reduced activation was also observed in the LH temporal type I source at 900–1200 ms [F(1, 11) = 7.37, p < 0.05]. In contrast, in the LH and RH frontal sources, activation increased from the 1^st^ to the 4^th^ presentation of the items at 300–1200 ms [LH: F(1, 10) = 9.5, p < 0.05; RH: F(1, 8) = 16.6, p < 0.01]. This increase was stronger for foreign than native language [LH: interaction Language x Stimulus familiarity, F(1, 10) = 5.2, p < 0.05]. In paired comparisons, the effect “familiar > new” was significant only for foreign language [LH: foreign t(10) = 4.3, p = 0.002; native t(10) = 1.3, p = 0.24; RH: foreign t(8) = 3.9, p = 0.005; native t(8) = 0.42, p = 0.69; Bonferroni corrected alpha = 0.0125].

**Table 1:** Group-level source strength values (mean (sd)) during the learning phase and post-learning manipulations in time windows and source clusters displaying learning effects.

			LEARNING PHASE (REPETITION, NO NOISE)				INTERMEDIATE NOISE (SNR +10 dB)				HIGH NOISE (SNR +0 dB)				1-BACK CONTROL TASK				RECOGNITION TASK			
			Foreign		Native		Foreign		Native		Foreign		Native		Foreign		Native		Foreign		Native	
**Time window**	**Source cluster**	**N**	**New**	**Fam**.	**New**	**Fam**.	**New**	**Fam**.	**New**	**Fam**.	**New**	**Fam**.	**New**	**Fam**.	**New**	**Fam**.	**New**	**Fam**.	**New**	**Fam**.	**New**	**Fam**.
**300–600 ms**	**LT Type II**	**11**	9.7	6.1	11.1	7.3	7.0	6.4	8.6	7.9	6.9	5.7	6.5	6.4	5.1	4.5	6.5	4.7	2.2	2.1	3.4	2.6
			(9.1)	(7.3)	(9.5)	(8.3)	(10.9)	(8.1)	(9.4)	(13.3)	(11.6)	(8.7)	(9.6)	(8.2)	(7.5)	(6.8)	(6.8)	(6.3)	(3.8)	(3.0)	(4.6)	(4.2)
**900–1200 ms**	**LT Type I**	**12**	19.7	17.4	28.9	21.3	24.8	20.6	35.7	29.4	23.2	21.4	31.0	30.9	18.5	15.7	23.5	19.2	17.8	14.8	22.9	17.3
			(9.2)	(9.6)	(16.1)	(16.0)	(12.3)	(13.8)	(21.7)	(18.9)	(14.9)	(14.7)	(21.7)	(16.6)	(8.4)	(11.7)	(10.7)	(12.1)	(10.3)	(9.4)	(16.1)	(11.2)
**300–1200 ms**	**LF**	**11**	11.1	19.0	13.2	16.0	17.0	18.3	14.7	16.7	15.2	15.4	13.7	13.3	13.8	14.2	15.2	15.0	11.8	12.2	12.9	14.0
			(7.5)	(9.3)	(6.7)	(11.3)	(9.5)	(11.8)	(8.0)	(8.3)	(9.8)	(9.2)	(7.5)	(7.0)	(8.5)	(8.5)	(9.9)	(10.9)	(9.6)	(13.1)	(12.7)	(14.9)
	**RF**	**9**	7.4	12.9	10.1	10.9	11.0	14.1	15.1	15.6	12.5	11.4	13.0	14.5	8.6	8.0	11.4	11.4	9.0	10.5	11.7	12.4
			(5.9)	(8.3)	(4.7)	(5.6)	(7.1)	(8.7)	(5.5)	(6.5)	(10.5)	(9.9)	(5.0)	(5.8)	(7.4)	(6.0)	(5.8)	(6.7)	(5.4)	(6.8)	(6.3)	(7.2)

LT = left temporal cortex, LF = left frontal cortex, RF = right frontal cortex, N = number of subjects.

### Influence of task demands

**Fig 5:**
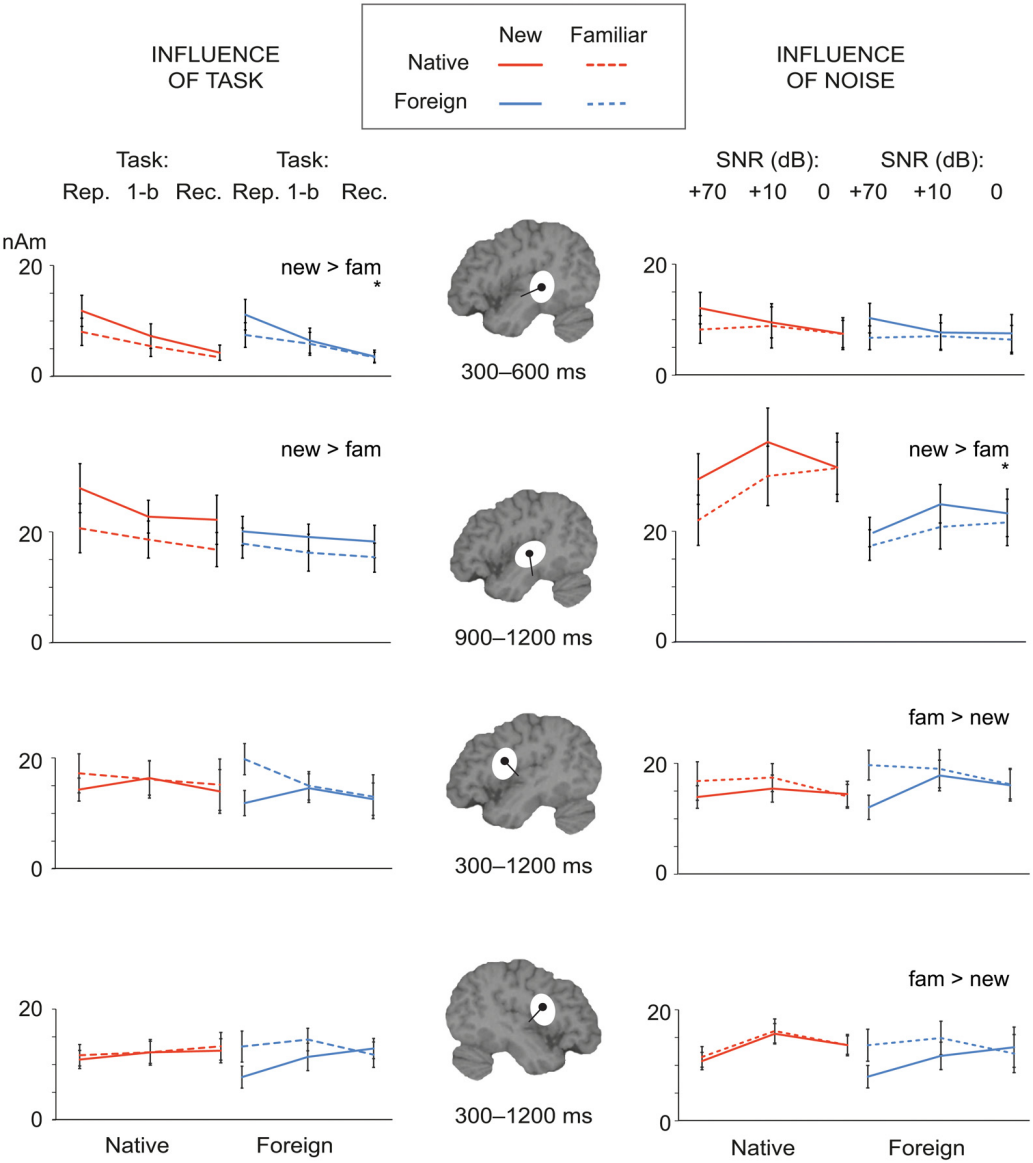
Group-level experimental effects during the post-learning manipulations. Summary of the mean activation strengths for native and foreign language during each of the tasks (*left*; repetition in the learning phase, 1-back control task, recognition) and noise levels (*right*; SNR +70 dB (no noise), +10 dB, 0 dB) in the left temporal and bilateral frontal sources. The time windows are the ones that showed learning effects during the initial learning phase. Statistically significant main effects of stimulus familiarity across experimental conditions (tasks / noise levels) and languages in the ANOVAs are denoted as text beside each plot (“new </> fam”, p < 0.05). Asterisks (*) below the text denote statistically significant interactions of stimulus familiarity and task / noise level.

Fig 5 summarizes the effects of the different post-learning manipulations on the effects in those time windows that displayed significant learning effects during initial the learning phase (Fig 4). Source strengths in the different conditions, in the cortical areas and time windows displaying learning effects, are presented in Table 1.

For the LH temporal type II sources at 300–600 ms, the main effect of Stimulus familiarity was consistently significant over all three tasks [F(1, 9) = 5.6, p < 0.05], but was somewhat reduced across tasks [overt repetition > 1-back control > recognition; interaction Task x Stimulus familiarity, F(2, 8) = 4.8, p < 0.05] (Fig 5, left). Also, the response strength was overall reduced from the learning phase to recognition [overt repetition > 1-back control > recognition; F(2, 8) = 5.6, p < 0.05].

For the LH temporal type I sources the effect of Stimulus familiarity at 900–1200 ms remained significant over all three tasks [F(1,10) = 23.7, p < 0.01], and there was no significant interaction of Task and Stimulus familiarity [F(2,10) = .75, p = 0.87]. The overall level of the responses was higher for native than foreign language when all three tasks were added into the analysis [main effect of Language at 900–1200 ms: F(1,11) = 9.1, p < 0.5].

For the frontal sources, the main effect of Stimulus familiarity (increase in sustained activity for previously heard items at 300–1200 ms) did not remain significant when all tasks were added into the analysis [LH: F(2,9) = 3.9, p = 0.076; RH: F(1,8) = 3.5, p = 0.099]. The effect was reduced in the 1-back and recognition tasks compared to the overt repetition task (without noise); the interaction Stimulus familiarity x Task reached significance in the LH [F(2,8) = 7.0, p < 0.05; RH: F(2,8) = 4.4, p = 0.058].

### Influence of noise

The effect of Stimulus familiarity in the LH temporal type II sources at 300–600 ms did not remain significant in noise [F(2,9) = 3.7, p = 0.083] (Fig 5, right). This response was overall attenuated by the added noise [F(2, 9) = 11.59, p < 0.01].

In contrast, in the left temporal type I sources at 900–1200 ms the main effect of Stimulus familiarity was consistently significant when all noise levels were included in the analysis [F(1, 11) = 21.4, p < 0.01]. The effect was strongest at the intermediate levels of noise (SNR +10 dB), but was diminished for the lowest SNR (0 dB) [interaction Stimulus familiarity x Noise, F(2, 10) = 5.3, p < 0.05; paired comparisons of familiar and new stimuli (across languages), no noise: t(11) = 2.7, p = 0.02; SNR +10 dB: t(11) = 3.0, p = 0.012; SNR 0 dB: t(11) = 0.8, p = 0.43; Bonferroni corrected alpha = 0.0167]. Overall, the LH temporal responses were stronger for native than foreign language when all three noise conditions were included in the analysis [main effect of Language: F(1,11) = 9.7, p < 0.01]. In paired comparisons, the activation was stronger for Finnish than Korean at both levels of added noise [no noise: t(11) = 2.1, p = 0.057; SNR +10 dB: t(11) = 2.8, p = 0.016; SNR 0 dB: t(11) = 3.4, p = 0.006].

For both LH and RH frontal responses, the main effect of Stimulus familiarity at 300–1200 ms remained statistically significant over all noise levels [LH: F(1, 10) = 11.5, p < 0.01; RH: F(1, 8) = 8.2, p < 0.05]. The interaction Stimulus familiarity x Noise did not reach significance in either hemisphere [LF: F(2, 9) = 3.4, p = 0.082; RF: F(2, 7) = 2.6, p = 0.14]; however, in paired comparisons (across languages) the familiarity effects were significant only in the no-noise condition [LF, no noise: t(10) = 3.1, p = 0.012; SNR +10 dB: t(10) = 1.3, p = 0.22; SNR 0 dB: t(10) = 0.13, p = 0.90; RF, no noise: t(8) = 3.6, p = 0.007; SNR +10 dB: t(8) = 1.3, p = 0.22; SNR 0 dB: t(8) = 0.12, p = 0.91; Bonferroni corrected alphas = 0.0167].

### Correlations of learning effects with behavioral measures

**Fig 6:**
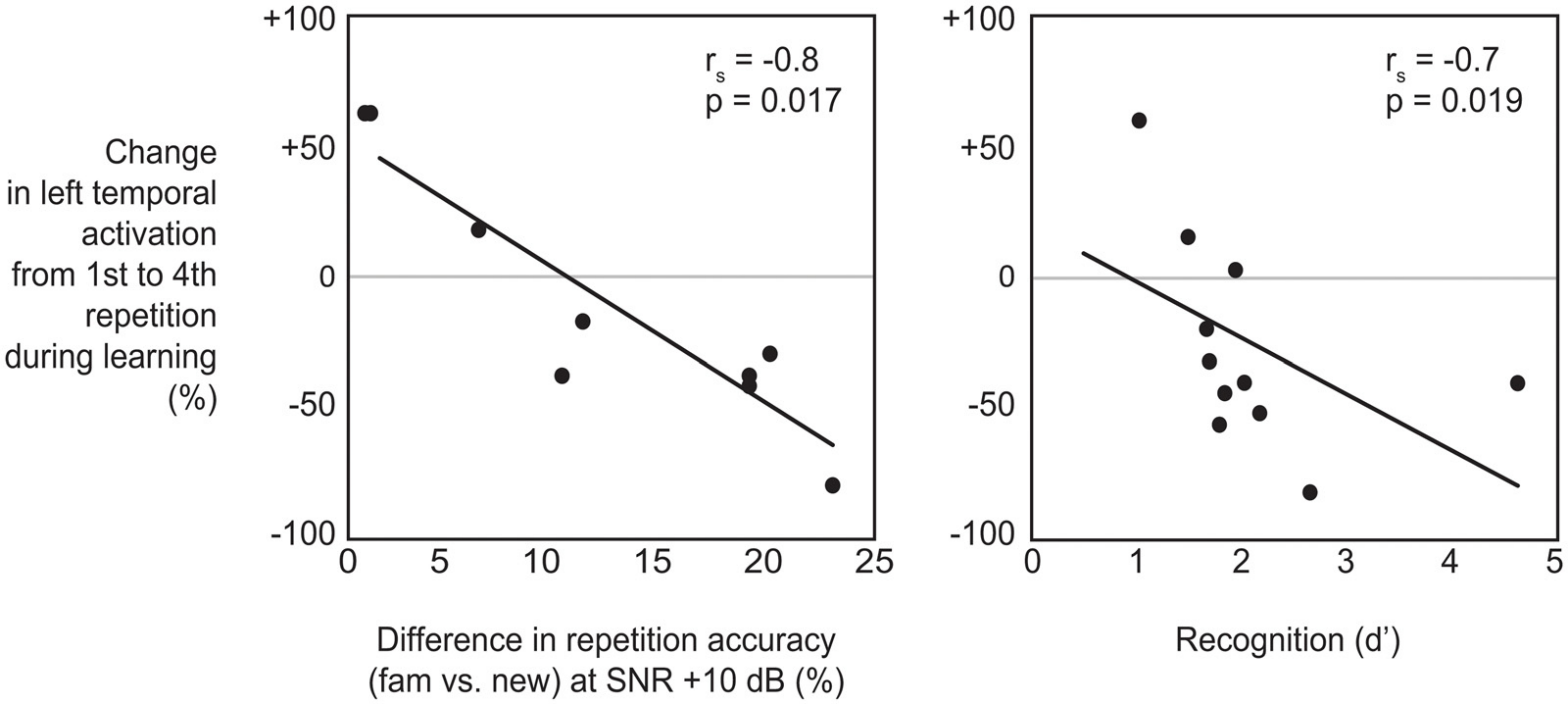
Significant correlations of neural effects and behavioral measures. Scatterplots of percent change of the left temporal activation from the 1^st^ to the 4^th^ repetition during learning as a function of percent difference in repetition accuracy between new and familiar word forms at SNR +10 dB (*left*) and as a function of recognition measure d’ (*right*).

The amount of signal change in the left temporal type I sources at 900–1200 ms during the learning phase (from the 1^st^ to the 4^th^ repetition) correlated significantly with the behavioral difference in repetition accuracy between new and familiar word forms in intermediate noise [ρ_s_ (Spearman’s rho) = 0.80, p = 0.017] and with recognition accuracy as measured by the sensitivity index d’ [ρ_s_ (Spearman’s rho) = 0.70, p = 0.019]. The more the left temporal signal strength had decreased during learning, the better the participant was at repeating the familiar items in noise and recognizing them in the recognition task (Fig 6). Signal change in other cortical sources did not significantly correlate with behavioral measures.

## Discussion

In the current study, we identified cortical effects related to incidental learning of native and nonnative spoken word forms and then investigated the functional roles of these effects using different post-learning manipulations. We first familiarized participants with a set of novel native and foreign spoken phonological forms in an incidental learning phase using overt repetition. We identified cortical effects reflecting learning of the new word forms. We then investigated the nature of those neural effects with two types of post-learning manipulations. To determine whether the frontal effects were functionally related to planning for overt articulation, a modified 1-back task and a recognition task were employed. The modified 1-back task involved articulation unrelated to the heard stimuli whereas the recognition task involved no articulation but required access to newly formed memory representations. Overt repetition with two levels of added noise was additionally administered to examine whether increased reliance on existing word-form level and/or native language phonotactic representations modulates the effects. Higher noise levels were assumed to trigger greater reliance on memory representations to compensate for the poor auditory signal.

The results indicate a functional dissociation between the temporal and frontal activations in phonological learning: sustained activation in the left planum temporale at 900–1200 ms showed reduced activation for repeated items in all tasks, likely reflecting activation of memory representations for the newly-learned word forms. In contrast, increased activation for familiar word form stimuli in the left and right premotor cortex at 300–1200 ms was seen only when planning for overt articulation, consistent with the hypotheses relating them directly to articulation. Dependence on existing word form representations in intermediate noise was seen in the learning effect in the left temporal but not in the frontal responses.

### Two types of learning effects in the left temporal and bilateral frontal cortices revealed in the learning phase

Incidental acquisition of novel word form representations was evidenced by participants’ improved repetition and recognition accuracy, especially for the native language. For the foreign language, the influence of stimulus familiarity on overt repetition was most evident when the items were embedded in added noise; overall, improvement through repetition was less substantial and recognition less reliable than for the native language. Because of technical problems in the behavioral data collection, behavioral results were available only from a subset of the participants, which could have affected the detection of familiarity effects. However, the observed neural effects—decrease of activation in two separate source clusters in the left temporal cortex and increase of frontal activation—replicated our earlier MEG results [10], and their source areas are in line with hemodynamic findings [8,9,11]. The present MEG study displayed an even higher cortical sensitivity to word-form learning than our previous study, presumably owing to the slightly higher number of participants and the meticulous acoustical matching of stimuli required by the parametric noise manipulation.

In Nora et al. [10], learning effects in the sustained temporal type I and frontal responses persisted after a consolidation phase including sleep, whereas the effect in the transient type II temporal response did not. In the present study, the learning effect (familiar < new) in the transient type II temporal response at 300–600 ms was somewhat reduced by the post-learning task manipulations, although consistently significant. Brain responses in this time window, distinguishable from the well-known N400 lexical-semantic response [60,61], have been implicated in mapping of auditory input to phonology [66,67]. The effect observed in the current study might be sensitive to the need for acoustic-phonetic analysis of the input for subsequent articulation. However, this effect was abolished in noise, when there was less acoustic detail available in the input signal, yet overt output was needed. Therefore, the learning effect observed in the temporal type II response may well reflect within-session repetition priming in acoustic-phonetic processing, in line with our previous results [10]. In the following sections, our focus will be on the sustained temporal type I activations and the frontal sustained activations.

### Frontal responses appear to reflect articulatory learning and increased effort in articulatory preparation

The increased frontal premotor activation for familiar versus new word forms was only observed in tasks that required translation of the heard stimulus into a corresponding motor program for overt output. This is consistent with the hypothesis that the frontal learning effects are associated with articulatory processing of the newly learned word forms: The frontal activation reacted to stimulus familiarity only when the specific gestural scores for the novel word forms had to be retrieved for overt output. Thus, it does not seem likely that the observed frontal effects would reflect successful recognition [68] or working memory maintenance [69], as these processes are engaged also in the 1-back and recognition tasks which did not show such familiarity effects. The current results are in line with earlier studies of speech perception, which have found that the motor system is more involved in speech perception during tasks requiring phoneme segmentation [70,71], and when overt or covert repetition rather than passive listening is involved [37,72]. A recent study fractionating the processes involved in pseudoword repetition found premotor activation for articulatory sequencing of phonological inputs during overt repetition, but not when processing the same stimuli in a 1-back matching task [73]. Premotor activation has been found to be scaled to articulatory complexity during trials involving both perception and production, but not during passive perception [25]. In a recent study [16], increased left frontal motor area activity for newly learned pseudowords was observed in a post-learning test without overt repetition, provided that the preceding learning phase had involved repetition; however, effects of tasks with differing articulatory demands and opportunities for covert articulation were not tested. In the present study the frontal activation seemed to reflect the new/familiar status of the word forms only when the perception of the word forms was immediately followed by production.

The observed learning effects in the frontal cortices were stronger for the foreign than native language. This result is in line with previous studies showing that non-native speech sounds activate the cortical motor regions more strongly than native speech sounds during passive listening [74], phoneme identification [75] and pseudoword repetition [12], and that foreign phoneme contrasts elicit stronger responses over frontal regions in a mismatch paradigm [76]. In previous studies investigating repetition of novel native pseudowords, decreased hemodynamic responses in premotor regions were observed for repeated exposure. These were interpreted to reflect more efficient representation of the articulation patterns (see e.g. [11]). In contrast, studies using artificial languages with nonnative syllable structure along with explicit segmentation tasks [14,38–40] or passive listening tasks [13] have found increased frontal activation for novel words that were learned from a continuous speech stream. These results were taken to indicate that motor regions mediate segmentation of novel speech stimuli.

Our current finding of increased frontal activation when learning novel word forms through overt repetition is consistent with increasing articulatory specification. The increase in response strength, especially for stimuli with unfamiliar phonology and phonotactic rules, may reflect extra effort needed for the online construction of the gestural scores for foreign word form articulation. More specifically, the observed effect might be related to ordering of the foreign acoustic material for overt output [77,78], as the input does not conform to native language syllables that can be processed as larger chunks during motor planning [79]. The internal motor representations for foreign language are, by necessity, flawed, as they have to be constructed, at least to some extent, from the phoneme and syllable representations for the native language. Updating the articulatory representations and correcting the subsequent motor output to more accurately match the input is thus more difficult for the foreign than native language. This could show up as a greater corrective command within the internal feedback control system for the newly-learned foreign word forms than the native pseudowords [79,80]. The role of the frontal reactivity as a corrective signal during construction of segmentational articulatory representation is supported by its early timing (from 300 ms onwards). Taken that no dramatic improvement in the overt reproduction of recurring foreign word forms occurred during learning, this corrective signal could have grown especially prominent across the repeated exposure to the foreign word forms. In contrast, reproducing the native pseudowords consisting of familiar syllables was quite easy and overt repetition performance during learning approached ceiling. This would have resulted in less need to correct the articulatory plans than for the foreign word forms.

The present pattern of results suggests that the signal increase for familiar compared to new items observed in the frontal responses reflects articulatory preparation for producing the new word forms. This articulatory processing seems to involve premotor regions bilaterally (no significant laterality effect), contrasting with the prevailing view that attributes formation of articulatory plans solely to the left hemisphere [15,81,82]. However, recruitment of right frontal areas has been observed when processing a less familiar language [83], in addition to the spatially separated left frontal activation for processing late-learned vs. native language [84]. Furthermore, responses in bilateral premotor areas are increased when preparing to produce more complex syllable sequences [85]. When right frontal activation has been observed during speech perception, it has been thought to reflect recruitment of additional domain-general processes for increasingly effortful manipulation of verbal material in working memory [86] or increased attentional focus on nonlinguistic perceptual aspects of language [87]. However, recent studies using intracranial recordings or transcranial magnetic stimulation have reported right-hemisphere participation also in auditory-motor transformations during pseudoword repetition [88] and speech motor control during naming [89].

### Left temporal responses are related to establishing new word form representations and activation of the phonological neighborhood

In contrast to the sustained frontal effects, the learning-related decrease in the sustained left temporal responses was observed regardless of whether the task required overt output or not. This suggests that the effect is not related to either internal monitoring of the planned overt output [90] or more efficient transfer of information to motor output [8], as has been suggested based on earlier fMRI results. Instead, the effect seems to reflect the availability of existing word-form level phonological representations to support processing; the amount of decrease in left temporal sustained response strength during learning correlated with the participants’ subsequent repetition accuracy in noise as well as their recognition accuracy.

Overt repetition of word forms in noise sought to probe how the newly-established word-form representations might facilitate perceptual performance. The behavioral results confirmed that the established word-form representations served to improve performance also in noisy conditions, as familiar word forms were repeated more accurately than new word forms. More pronounced temporal or frontal cortical learning effects in noise can be assumed to point to retrieval of the newly-learned word-form level auditory or motor representations from memory during processing of the new word forms. We expected especially the frontal effects to be enhanced in noise, since articulatory processes have been suggested to facilitate perception in demanding conditions [46]. However, in the present study, although the main effects of stimulus familiarity in the frontal premotor cortices were consistently significant in noise, they tended to decrease, and paired comparisons showed a significant effect of familiarity only in the no-noise condition. This may have been because mapping from the impoverished auditory signal to the newly established articulatory patterns was unreliable. Instead, the observed learning effect in the left temporal sources seemed to remain at the same level or was possibly even somewhat enhanced in intermediate noise. At the highest noise level, where on average only 4 in 100 of all stimuli could be accurately reproduced, the effect of stimulus familiarity was diminished. This is in line with previous studies showing that the effects of noise on cortical responses to speech stimuli may be nonlinear [45,91,92]. The findings of the current study thus suggest that the influence of familiar phonological word-form representations on input processing might especially appear in regions involved in auditory processing, but the accessibility of recently learned forms for articulatory planning is less clear in noise.

In the current study, we investigated learning and retrieval of new meaningless word forms. Learning meaningful words might engage the frontal regions differently. Effects related directly to successful word retrieval have been observed in ventral frontal activation, apparently functionally distinct from the premotor activation observed in the current study [68,93–96]. Also, increased frontal activation has been observed in processing of familiar word- and sentence-level stimuli in adverse conditions [44]. Thus, the current results do not exclude the possibility that in the case of meaningful lexical stimuli, retrieval of prior knowledge from memory and its integration with sensory information during input processing could be reflected also in the frontal areas that are part of the ventral route of speech processing [97,98].

Contrary to our hypotheses, we did not observe any of the effects of word form familiarity to be enhanced in noise for the native language stimuli more than for the foreign language stimuli, a finding that could have indicated top-down effects from familiar phonotactics to support retrieval of the newly-learned word forms. However, the left temporal sustained response did display generally stronger activation for native than foreign language in noise. Previous studies have linked the overall increased activation for stimuli embedded in noise to top-down mechanisms that enhance speech perception in demanding conditions [45,72,91,99,100]. The increased response strength observed for the native language in noise could also be related to automatic activation of the lexical neighborhoods of the novel word forms [101–103], for both newly-learned and new pseudowords, and thus does not necessarily directly reflect the quality of the memory representations for the heard novel words. Phonotactically legal pseudowords have been reported to elicit a stronger N400 response compared to phonotactically illegal nonwords or foreign words [102,104,105], probably because they are treated as possible word candidates and trigger lexical search to a larger extent (for a review see [106]). This activation of lexical neighbours might be even more substantial in noise, when pseudowords are not easily distinguished from real native words [44]. Indeed, substitution errors indicated that more of the native pseudowords were mistaken for real native words when presented in noise than in the no-noise condition. However, it cannot be ruled out that the observed language difference might be, at least partially, related to differences between the two languages, which may also be differently affected by the addition of noise.

## Conclusions

The present results revealed a functional dissociation between temporal and frontal activations in learning and processing phonological forms. During auditory processing, the sustained temporal responses appear to reflect influences of the acquired word-form representations regardless of task demands, and are sensitive to increased reliance on such representations when the sensory input is degraded. This is in line with a view suggesting that the identification of spoken words relies on prediction of the upcoming speech segments based on the established word-form representations, with the superior posterior temporal cortex coding the mismatch between these expectations and auditory input [97,103,107–109]. In contrast to the left temporal effect, the frontal effects seem to be related to establishing articulatory representations for the new word forms, as they were only detected in conditions requiring overt output, and were not preserved in degraded sensory input.
